# Soluble Megalin is Reduced in Cerebrospinal Fluid Samples of Alzheimer’s Disease Patients

**DOI:** 10.3389/fncel.2015.00134

**Published:** 2015-04-15

**Authors:** Carlos Spuch, Desireé Antequera, Consuelo Pascual, Soledad Abilleira, María Blanco, María José Moreno-Carretero, Jesús Romero-López, Tetsuya Ishida, Jose Antonio Molina, Alberto Villarejo, Felix Bermejo-Pareja, Eva Carro

**Affiliations:** ^1^Department of Neurology, Instituto de Investigación Biomédica de Ourense, Pontevedra y Vigo (IBI)/Xerencia de Xestión Integrada de Vigo-SERGAS, Vigo, Spain; ^2^Center for Biomedical Research in Neurodegenerative Diseases (CIBERNED), Madrid, Spain; ^3^Neuroscience Group, Research Institute Hospital, Madrid, Spain; ^4^Department of Histology and Cell Biology, School of Medicine, Kagawa University, Miki, Japan; ^5^Service of Neurology, Research Institute Hospital, Madrid, Spain

**Keywords:** megalin, Alzheimer’s disease, cerebrospinal fluid, choroid plexus, epithelial cells, amyloid β, clearance, patients

## Abstract

Megalin or low-density lipoprotein receptor-related protein-2 is a member of the low-density lipoprotein receptor family, which has been linked to Alzheimer’s disease (AD) by clearing brain amyloid β-peptide (Aβ) across the blood–cerebrospinal fluid barrier at the choroid plexus. Here, we found a soluble form of megalin secreted from choroid plexus epithelial cells. Soluble megalin levels were also localized in the human cerebrospinal fluid (CSF), being reduced in AD patients. We have also shown that soluble megalin binding to Aβ is decreased in the CSF of AD patients, suggesting that decreased sequestration of Aβ in the CSF could be associated with defective clearance of Aβ and an increase of brain Aβ levels. Thus, therapies, which increase megalin expression, at the choroid plexus and/or enhance circulating soluble megalin hold potential to control brain Aβ-related pathologies in AD.

## Introduction

Megalin, a transmembrane glycoprotein and member of the low-density lipoprotein (LDL) receptor-related family, is an endocytic receptor expressed on the apical surface of several epithelial cells that internalizes a variety of ligands including nutrients, hormones and their carrier proteins, signaling molecules, morphogens, and extracellular matrix proteins. In adults, megalin expression is restricted to the central nervous system (CNS), the choroid plexus (Chun et al., 1999; Carro et al., 2005), the ependymal cells of the lateral ventricles (Gajera et al., 2010), and to the spinal cord (Wicher et al., 2006). Megalin has a relatively large extracellular domain, responsible for ligand binding, a small cytoplasmic domain, and a single transmembrane domain (Marzolo et al., 2003). Based on the important roles of its ligands and its tissue expression pattern, megalin has been recognized as an important component of many pathological conditions, including Alzheimer’s disease (AD). Megalin is required for efficient brain Aβ clearance, mediating the transport of Aβ from the cerebrospinal fluid (CSF) into the choroid plexus epithelium (Hammad et al., 1997; Alvira-Botero and Carro, 2010; Pascale et al., 2011). Megalin is also expressed in neurons, where it interacts with APP and FE65, participating in the neurite branching. In AD, this interaction has been demonstrated to be important in Aβ-mediated neurotoxicity (Alvira-Botero et al., 2010).

Another member of LDL receptor-related family, the low-density lipoprotein receptor-related protein 1 (LRP-1), has been mainly implicated in blood–brain barrier (BBB) Aβ clearance (Bell et al., 2007), but it has also been reported to be expressed in the choroid plexus (Kounnas et al., 1994), where it is involved in the clearance of Aβ in the CSF. LRP-1 is a substrate for both β-secretase (BACE) and γ-secretase (Von Arnim et al., 2005). BACE cleaves the N-terminus extracellular domain of LRP-1, which releases soluble LRP-1 (Von Arnim et al., 2005), implying circulation of Aβ regulation, and possibly Aβ-related brain pathology (Sagare et al., 2007).

Megalin has also been demonstrated to be released into the extracellular medium (Bachinsky et al., 1993; Spuch et al., 2010). These soluble forms are fragments of approximately 200–220 kDa, suggesting that they are truncated forms of the membrane-bound megalin. Indeed, the transmembrane domain of megalin is also a substrate for the γ-secretase complex (Zou et al., 2004; Biemesderfer, 2006). Megalin has also been found in urine-derived exosomes (Pisitkun et al., 2004). It is therefore possible that under some pathophysiological conditions, the levels of megalin associated with exosomes or megalin derived from protease-mediated shedding of the extracellular domain could be affected and could serve as a diagnostic biomarker. In addition, the secretion of soluble forms of megalin could be a physiological mechanism to regulate the availability of ligands and/or to transport ligands. Thus, the secretion of soluble fragments of megalin into the CSF could signify a relevant mechanism controlling the availability of the receptor, storage of the ligands in the CSF, and its function at the blood–CSF barrier.

Taking these data together, we hypothesize that megalin could also be proteolytically processed by the choroid plexus epithelial cells, which would lead to the release of a secreted form of megalin into the medium. In this study, we find that a soluble form of megalin exists in the CSF, and its levels are decreased in AD patients, compromising brain Aβ clearance.

## Materials and Methods

### Human sample

We included three groups of subjects: (1) AD patients; (2) Parkinson’s disease (PD) patients; and (3) elderly non-demented controls. For AD patients, the diagnosis was established according to the National Institute on Neurological Disorders and Stroke, and the Alzheimer’s Disease and Related Disorders Association (NINDS–ADRDA) guidelines (McKhann et al., 2011). Parkinson’s patients were diagnosed according to the criteria of probability (Gelb et al., 1999). Disease severity was evaluated using mini-mental state examination (MMSE) scores (Table 1). Inclusion criteria for cognitively normal older individual subjects were MMSE scores of 24–30, with no history or clinical signs of neurological or psychiatric disease or cognitive symptoms. Approval for sample ascertainment and collection was obtained from the Ethics Committee of the Hospital “12 de Octubre.” Written informed consent was obtained from all participants or representatives prior to their participation.

**Table 1:** **Demographic characteristics of patients with neurodegenerative diseases and control subjects**.

	AD patients	PD patients	Control subjects	*p* Value
Average age	76.1±1.4	67.7±1.6	67.5±1.9	NA
Gender (M/F)	14/15	19/13	14/10	NA
Mean MMSE	19.4±0.8*	28.0±0.5	30.0±1.5	*p* < 0.05*
Mean onset	2.5±0.2	4.1±0.8	nd	NA
CSF Aβ_42_ (pg/ml)	422.8±58.2*	nd	768.3±163.9	*p* < 0.05*
CSF Tau (pg/ml)	664.8±7.9**	nd	379.1±47.3	*p* < 0.001**

*MMSE, mini-mental state examination; CSF, cerebrospinal fluid; AD, Alzheimer’s disease; PD, Parkinson’s disease; NA, not applicable; nd, not determined*.

*Results are expressed as mean ± SEM*.

*p < 0.05.

**p < 0.001 versus control subjects.

A total of 85 CSF samples were included in this study from patients with AD, PD, as well as healthy control subjects obtained at the Hospital 12 de Octubre (Table 1). CSF collection was conducted following standard operating procedures. Briefly, 12 ml of CSF were collected in a polypropylene tube by lumbar puncture in the L3–L4 or L4–L5 interspaces and centrifuged at 2000 × *g* at 4°C for 10 min. The supernatant was aspirated, gently mixed to avoid possible gradient effects, and aliquoted into polypropylene tubes that were stored at −80°C for subsequent biochemical analyses.

Choroid plexus tissue was obtained from the Department of Pathology and Neuropathology (Xerencia de Xestión Integrada de Vigo-SERGAS), following the Spanish legislation guidelines. The samples analyzed for AD studies included choroid plexus from stages V–VI (*n* = 9, 76.4 ± 4.1 years), and age-matched controls (*n* = 6, 69.6 ± 3.1 years).

### Cell culture

Choroid plexus epithelial cell cultures were prepared as described previously (Carro et al., 2002). Choroid plexus were dissected from 3- to 5-day-old Wistar rats (Charles River). Choroid plexus epithelial cells were seeded on laminin-coated 24-well plates with Dulbecco’s modified Eagle’s medium (Lonza) containing 10% fetal bovine serum (Lonza), 2 mM glutamine (Sigma), 100 U/ml penicillin (Lonza), and 100 μg/ml streptomycin (Lonza). Cells were grown to confluence for 5–7 days at 37°C in a humidified atmosphere containing 5% CO_2_, and serum starved for 2 h. Then, human oligomeric Aβ_40_ (5 μg/ml; AnaSpec, Inc., San Jose, CA, USA) was added. Aβ_40_ was previously dissolved in acetic acid 0.1M, and then dissolved in sterile distilled water as reported by Dietrich et al. (2008). At different times after stimulation, cell culture medium was collected, and cells were either fixed for immunocytochemical processing or homogenized for immunoblot determination.

### Measurement of extracellular/soluble megalin

Culture medium was replaced with fresh medium about 2 h prior to Aβ treatment. Cells were stimulated with 5 μg/ml of Aβ_40_ at 37°C for 24 and 48 h. Then, culture medium was collected and centrifuged at 2000 × *g* for 3 min to pellet any possible suspended cells or debris in the medium. The supernatant medium was collected, and stored at −80°C for immunoblot analysis or mass spectrometry.

### Immunoblotting and immunoprecipitation

Cell lysates and medium from choroid plexus epithelial cultures and SCF samples were analyzed by immunoblot. Protein content was determined by the DC method (Bio Rad Laboratories, Inc.). Equal amounts of protein (50 μg/lane) were boiled and separated by SDS-PAGE (5–20%), and transferred onto PVDF membranes (Millipore). Non-specific binding was blocked by incubation in 5% non-fat milk in Tris-buffered saline (100 mM NaCl, 10 mM Tris, pH 7.4) containing 0.2% Tween (TTBS) for 1 h at room temperature. Afterwards, membranes were incubated overnight at 4°C with different antibodies in TTBS with 3% bovine serum albumin. We used one specific homemade antibody that recognizes the specifically soluble megalin described by Ishida et al. (2006). The antibody anti-megalin (mAb2E12) recognizes a soluble 210-kDa molecule lacking a cytoplasmic domain, which corresponds with a truncated form of membrane-bound megalin. For the truncated form of LRP1 (85 kDa), we used the anti-LRP1 antibody (EPR3724, Abcam) corresponding to aa 4450 to the C-terminus of LRP1 and truncated form of LRP1. Protein loading was monitored using a mouse monoclonal antibody against β-actin (1:10000, Sigma-Aldrich, MO, USA).

The interaction of Aβ with megalin and LRP-1 was assessed by co-immunoprecipitation. In these experiments, megalin or LRP-1 was immunoprecipitated from CSF by incubating 500 μl of CSF sample with either anti-megalin (1:100, Santa Cruz) or anti-LRP (1:200, Abcam) antibodies, on a rotating wheel overnight, followed by the addition of protein A-agarose and an additional incubation for 2 h. The immunocomplex was subsequently washed twice with washing buffer (20 mM Tris, pH 7.4, 150 mM NaCl). Thirty microliters of volume samples including loading buffer were boiled for 5 min. Then, they were resolved on SDS-polyacrylamide gel, and transferred onto a PVDF membrane. Following incubation with phosphate buffer (PB 0.1M supplemented with 0.1% Tween-20, containing 5% low fat milk to block non-specific binding sites), membranes were incubated with anti-Aβ antibody (1:500, MBL, Nagoya, Japan) at 4°C overnight, and then with a secondary antibody conjugated with horseradish peroxidase (Bio-Rad). The immunoreactive signal was revealed in a chemiluminiscent substrate detection system ECL (Bio-Rad). Protein band densities were quantified using ImageJ software (NIH, Bethesda, MD, USA) after scanning the images with ImageQuant software (GE Healthcare). To verify the protein–protein interaction, Aβ was immunoprecipitated by incubating with anti-Aβ antibody (1:500, MBL, Nagoya, Japan), and the co-immunoprecipitated proteins were identified on a Western blot with anti-megalin (1:5000, Abcam), and anti-LRP (1:20000, Abcam). In addition, the amount of proteins immunoprecipitated by their own primary antibodies was routinely measured on Western blots.

### Mass spectrometry

Culture medium samples from choroid plexus epithelial cell culture were loaded on a SDS-PAGE 4–20% gel. A protein band matching the molecular weight of megalin was excised from the gel and destained. In-gel digestion was carried out sequentially with trypsin and endopeptidase Asp-N for 16 h each. To validate the presence of soluble megalin in culture medium, this protein was identified by a MALDI-TOF/TOF mass spectrometer 4800 Proteomics Analyzer (Applied Biosystems, Framingham, MA, USA) and 4000 Series Explorer™ software (Applied Biosystems).

### Immunocytochemistry

The non-treated and Aβ_40_-treated choroid plexus epithelial cells were fixed with 4% formaldehyde in PB (pH 7.4) at room temperature for 30 min. Cells were then stained with anti-megalin (mAb2E12, 1:500, gift from Tetsuya Ishida, Kagawa University School of Medicine, Japan) antibody at 4°C, overnight.

After the cells were washed three times with PB, secondary donkey anti-mouse IgG 488 (1:1000, FluoProbes^®^, Interchim) was added to the cells and incubated at room temperature for 1 h in the dark. The cells were then washed three times, sealed with VECTASHIELD (Vector Laboratories) and observed under a Zeiss LSM 510 Meta scanning laser confocal microscope (Leica Microsystems).

Levels of endogenous Aβ_42_, and tau in CSF samples were determined using the Aβ_42_ human ELISA Innotest kit (Innogenetics), and Tau human ELISA Innotest kit (Innogenetics), respectively.

### Statistical analysis

The Kolmogorov–Smirnov *Z* test was used to analyze the distribution of the samples. For normally distributed data, statistical significance was tested using Student’s *t*-test when comparing two groups, and a two-way ANOVA followed by *post hoc* Mann–Whitney test was used for multiple comparisons. All calculations were made using SPSS v15.0 software. Statistical significance was set at *p* < 0.05.

## Results

### Aβ_40_-induced decrease in extracellular megalin

**Figure 1:**
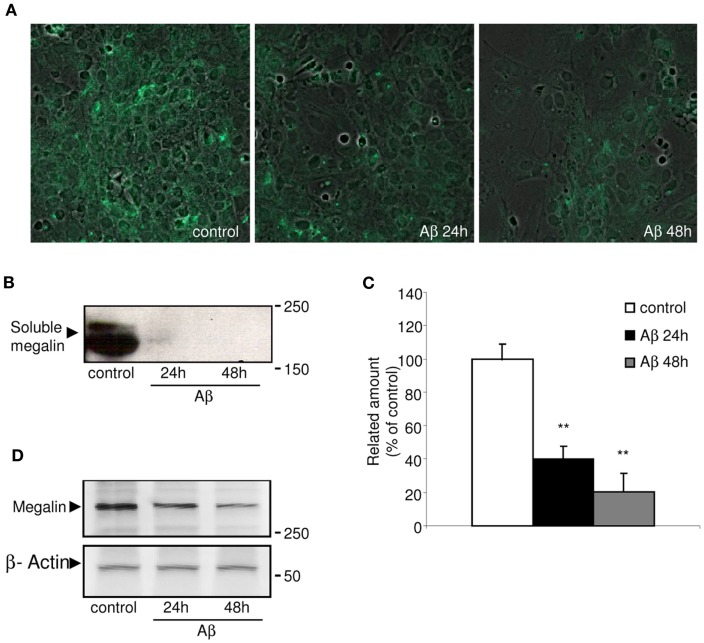
**Immunolocalization of soluble megalin in cultured choroid plexus**. **(A)** The green fluorescence represents megalin signal in the culture medium from choroid plexus epithelial cell cultures. The amount of megalin in the culture media from these cells was markedly decreased following Aβ_40_ treatment for 24 and 48 h. **(B)** Culture media were resolved on SDS-PAGE, and the amount of extracellular megalin after Aβ_40_ treatments was measured on Western blots. **(C)** The histogram represents densitometric values from three replicates. **(D)** A representative Western blot showing reduced expression of membrane-bound megalin in cell lysates following Aβ_40_ treatment for 24 and 48 h. Results are expressed as mean ± SEM. **p < 0.01.

Megalin is known to be secreted into the extracellular matrix as a soluble form, although the mechanism(s) and molecular pathways(s) underlying its secretion have not been identified. Figure 1A shows the immunocytochemistry results, in which choroid plexus epithelial cells were stained with soluble megalin antibody. Under non-stimulated conditions, soluble megalin was detected in the extracellular space. We further investigated the possibility that Aβ could alter the levels of megalin in the culture medium. As shown in Figure 1B, the immunoblotting of soluble megalin showed a band (at approximately 210 kDa), corresponding with the extracellular form of megalin (Ishida et al., 2006). To substantiate this, the band corresponding to soluble megalin was excised and analyzed by mass spectrometry after an in-gel digestion. The soluble megalin band was further confirmed, with amino acid coverage of 34%.

Because megalin has a greater affinity for Aβ_40_ than for Aβ_42_ (Deane et al., 2004), we used the former, more soluble form of Aβ_40_ in this study. Soluble megalin levels were significantly decreased in the culture medium of choroid plexus epithelial cells both 24 and 48 h after Aβ_40_ exposure compared to vehicle-treated cell cultures (Figures 1B,C; *p* < 0.01). As expected, Aβ_40_ administration also induced an important reduction in expression of membrane-bound megalin in cell lysates (Figure 1D).

### Extracellular megalin in human CSF

**Figure 2:**
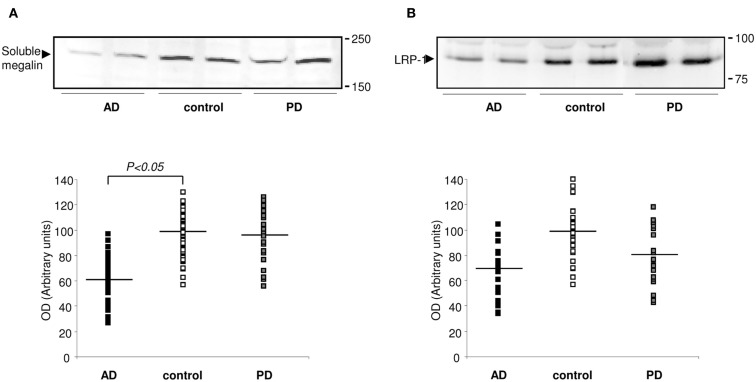
**Expression of megalin and LRP-1 in human CSF**. **(A)** Soluble megalin **(A)**, and LRP **(B)** expression measured on Western blots in CSF samples from non-demented subjects (controls; white), with Alzheimer’s disease (AD; black), and Parkinson’s disease (PD; gray). Scatter plots represent results from three replicates. Results are expressed as mean ± SEM. *p < 0.05.

The fact that megalin is expressed by choroid plexus epithelial cells, and is secreted into the extracellular medium, prompted us to examine CSF for its presence. Statistical analysis for age or gender revealed no significant differences between the four diagnostic groups (AD, PD, and controls). Baseline biochemical characteristics of subjects included in this study were also shown in Table 1. The mean MMSE (*p* < 0.05), tau (*p* < 0.01), and Aβ_42_ (*p* < 0.05) CSF levels were significantly different between AD group and control subjects (Table 1). Immunoblotting of human CSF indeed revealed a single band (210 kDa) that cross-reacted with mAb2E12 antibody, corresponding to megalin (Figure 2A). Notably, in AD patients, soluble megalin levels in the CSF were 40% lower than in non-demented subjects and PD patients (Figure 2A). Note that Aβ deposits were not detected in choroid plexus from PD (1.07 ± 0.02 pg/ml) or non-demented subjects (1.02 ± 0.05 pg/ml), whereas choroid plexus Aβ accumulations were significantly increased in AD patients (3.77 ± 1.22 pg/ml, *p* < 0.01 versus control group). These findings suggest that choroid plexus Aβ accumulation might induce a decrease in CSF soluble megalin, in accordance with previous *in vitro* data.

Whereas soluble LRP-1 normally circulates in plasma (Quinn et al., 1997), in this study, we reported that soluble LRP-1 was also detected in human CSF by Western blotting (Figure 2B). In AD patients, soluble CSF LRP-1 levels were low but not significantly lower than in non-demented controls (Figure 2B).

**Figure 3:**
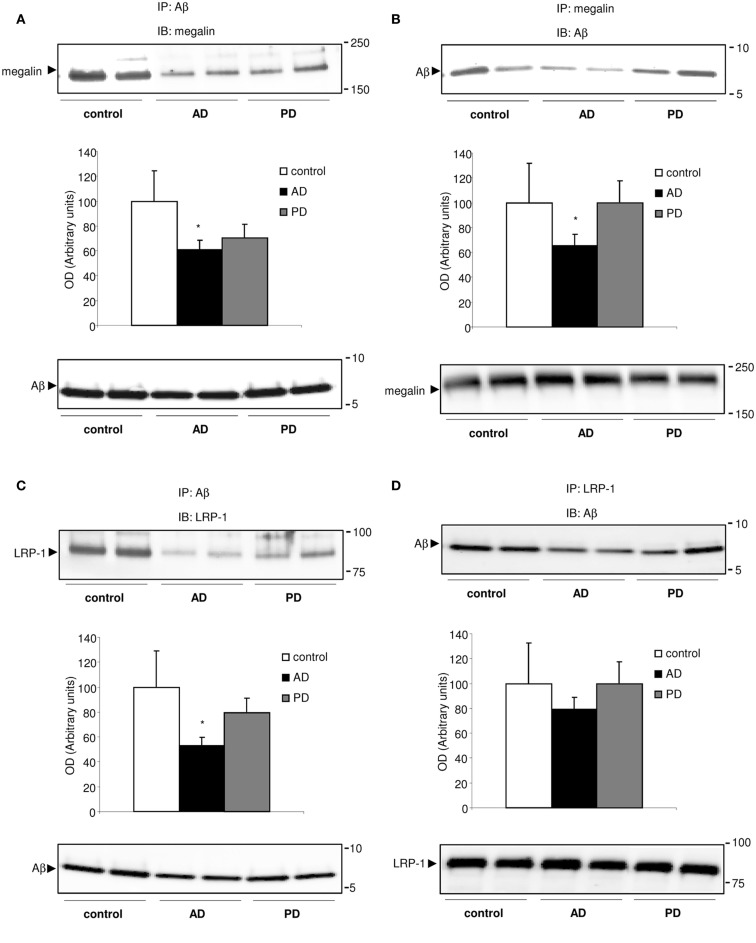
**Megalin-bound, and LRP-1-bound Aβ40 in human CSF**. **(A–D)** Co-immunoprecipitation of Aβ_40_ with megalin and LRP-1 in human CSF. CSF samples were subjected to immunoprecipitation (IP) with an anti-Aβ antibody **(A,C)**, followed by detection of megalin, and LRP-1 on Western blots (IB). Reversed immunoprecipitation with anti-megalin **(B)** and LRP-1 **(D)** antibodies followed by detection of Aβ with anti-Aβ antibody. Densitometric values for co-immunoprecipitated Aβ with megalin and LRP-1 were normalized, respectively, against that for each precipitated receptor. The histograms represent results from three replicates. Results are expressed as mean ± SEM. *p < 0.05. AD, Alzheimer’s disease; PD, Parkinson’s disease.

Because megalin binds Aβ (Hammad et al., 1997; Alvira-Botero and Carro, 2010), and soluble LRP-1 also is a major binding protein for Aβ (Sagare et al., 2007), we hypothesized that soluble megalin might also be involved in Aβ clearance in CSF. As shown in Figure 3, in co-immunoprecipitation experiments, a substantial amount of Aβ co-immunoprecipitated with both megalin and LRP-1 from control CSF samples. In CSF samples from AD patients, the amount of Aβ “pulled down” by megalin or LRP-1 was reduced (Figures 3A–C, upper panels). Although a similar amount of Aβ was precipitated (Figure 3C, down panel), significantly less megalin and LRP-1 were co-immunoprecipitated with Aβ in CSF samples from AD patients (Figures 3A,C, upper panels). This result was confirmed by a reversed immunoprecipitation experiment, in which anti-megalin and LRP-1 antibodies were used during precipitation, followed by detection of Aβ on Western blots with anti-Aβ antibody (Figures 3B,D). The reduced amount of Aβ co-immunoprecipitated with soluble forms of megalin and LRP-1 was not due to a decrease in the amount of megalin and LRP-1 precipitated by the antibodies, since similar amounts of megalin and LRP-1 were detected from the precipitated samples (Figures 3B,D, lower panels).

## Discussion

This study provides the first evidence for secreted soluble megalin from rat choroid plexus epithelial cells. We also found that megalin was present as a soluble receptor in CSF, possibly secreted by choroid plexus epithelial cells, in agreement with previous studies (Orlando and Farquhar, 1993; Kounnas et al., 1994).

Megalin is an endocytic membrane receptor of 600 kDa. It is a transmembrane glycoprotein that has a relatively large extracellular domain, a small cytoplasmic domain, and a single transmembrane domain that targets it to membrane domains rich in cholesterol and glycosphingolipids (Marzolo et al., 2003), and is a substrate for the γ-secretase complex (Zou et al., 2004; Biemesderfer, 2006). This last process involves proteolysis of the membrane protein, culminating in the release of the intracellular domain of megalin from the membrane (Zou et al., 2004). Previous studies have indicated that megalin exists in two forms: a membrane-bound form, and a soluble form (Bachinsky et al., 1993). Soluble forms of megalin have been demonstrated to be released into the medium of an immortalized rat proximal tubule cell line (Jung et al., 1998), the supernatant of a yolk sac carcinoma cell line (Orlando and Farquhar, 1993), into the endolymphatic sac (Ishida et al., 2006), and into normal human urine (Kounnas et al., 1994; Norden et al., 2002). In cultured epithelial cells of renal tubules that express megalin, both “soluble” and “insoluble” forms of the receptor have been reported (Bachinsky et al., 1993; Orlando and Farquhar, 1993). It has been suggested that under some pathological conditions, a soluble form of megalin receptor is produced by brain microvessels, choroid plexus, and/or ventricle ependyma and transported to neuronal targets. All these findings are consistent with a model of regulated processing of the receptor.

Soluble forms of megalin previously described (Bachinsky et al., 1993; Orlando and Farquhar, 1993; Kounnas et al., 1994; Norden et al., 2002; Ishida et al., 2006), and also showed in our study, are fragments of approximately 200–220 kDa, suggesting that they are truncated forms of the membrane-bound megalin. Some studies have shown that the ectodomain shedding of megalin is mediated by a metalloprotease in an opossum kidney proximal tubule cell line (May et al., 2002; Zou et al., 2004; Biemesderfer, 2006). Our hypothesis supports previous studies reporting that several soluble forms are produced by the release of the ectodomain via a single proteolytic cleavage of the membrane receptors, whereas others are secretory products.

Lipoprotein receptor-related protein 1, which also belongs to the LDL gene family, is also subjected to regulated intramembrane proteolysis (May et al., 2002). LRP-1 has been also reported to be a substrate for both BACE and γ-secretase (Von Arnim et al., 2005). BACE interacts with the intracellular domain of LRP-1, induces LRP-1 extracellular domain cleavage, and the subsequent release of the LRP-1 intracellular domain from the membrane. BACE activity at endogenous levels leads to an increase in LRP-1 C-terminal fragment, and to the release of secreted LRP-1 in the medium (Von Arnim et al., 2005). Since megalin is also a member of this gene family and closely related to LRP-1, we suggested that megalin could be proteolytically processed by similar mechanisms. Indeed, BACE is present and active in choroid plexus (Crossgrove et al., 2007; Dietrich et al., 2008).

The physiological significance of most soluble forms of the receptor is poorly understood, although it is known that many soluble forms have ligand-binding capacity. Preliminary data have already identified soluble LRP-1 in plasma (Quinn et al., 1997). Here, native soluble LRP-1 normally controls 70–90% of circulating Aβ in humans through peripheral binding, regulating Aβ metabolism and clearance under physiological conditions and in AD. Soluble LRP-1 function is compromised in AD, which may contribute to elevated brain Aβ (Sagare et al., 2007). In AD subjects, there was an important drop in soluble LRP-1-bound Aβ associated with a severe increase in free, protein-unbound Aβ (Sagare et al., 2007).

In a recent study with patients diagnosed with AD and diabetes mellitus type-1, high levels of soluble LRP1 in CSF were described compared with the control subjects (Ouwens et al., 2014). This discrepancy with our data may be due to insulin levels, as it has been reported that insulin promotes the synthesis of LRP-1 (Bell, 2012).

In the present study, we have detected both soluble megalin and LRP-1 in CSF samples. Reduced soluble megalin after Aβ exposure was not only detected in the choroid plexus culture medium but also was found in the CSF from AD subjects compared to non-demented controls, being absent in PD patients. We found that Aβ_40_ treatment reduces soluble megalin levels in cultured choroid plexus epithelial cells, suggesting that this extracellular megalin isoform might be reduced under Aβ accumulation conditions. This is in agreement with previous work showing that soluble LRP function is compromised in AD, and may contribute to elevated brain Aβ (Sagare et al., 2007). Taken into account that Aβ peptides are accumulated in the choroid plexus in AD patients (Dietrich et al., 2008), it might be suggested that Aβ initially causes a reduction in soluble megalin levels.

Since megalin and LRP-1 have been also reported to be involved in transport of Aβ from the CSF (Hammad et al., 1997), we next showed that both megalin and LRP-1 were the main binding proteins for Aβ in human CSF. After co-immunoprecipitation with megalin and LRP-1-bound Aβ, we found that in AD patients there is a reduction of binding affinity for Aβ with both soluble forms of megalin and LRP-1 in CSF. There was a clear trend for megalin- or LRP-1-bound Aβ to decrease in AD patients compared to controls, and PD patients.

It was described that in AD patients there is an oxidation of LRP-1, resulting in a reduction of binding affinity for Aβ (Owen et al., 2010). Although there is no evidence of similar megalin oxidation, we cannot discount its possibility, which would result in a reduction in Aβ clearance, and consequently, an increase in brain Aβ.

The presence of megalin has been described in urine-derived exosomes (Pisitkun et al., 2004; Fernandez-Llama et al., 2010). Because exosomes are secreted from almost all cells, it is also possible that in other organs, as well as the kidney, the release of exosomes containing megalin could be a physiological mechanism to regulate the availability of ligands and/or to transport ligands from one cell to another. Our present study did not discriminate between megalin associated with exosomes and megalin derived from protease-mediated shedding of the extracellular domain. It is therefore possible that under some pathophysiological conditions, the levels of megalin secreted in exosomes could be affected and could serve a diagnostic biomarker. However, until now, there has been no report indicating changes in the presence of megalin in exosomes in different diseases, such as AD, and further research will be required to understand this process.

In conclusion, in this study, we were able to identify soluble forms of megalin and LRP-1 in human CSF. We also propose that these soluble fragments could be used as biomarkers for AD diagnosis. Additionally, the complexes LRP1-Aβ and megalin-Aβ in CSF could be regulating the availability, storage, and clearance of Aβ from the brain to the blood. Thus, changes in the kinetic affinity binding probably increase brain Aβ levels and consequent Aβ-induced toxicity effects. It is possible that Aβ directly regulates soluble megalin levels, and subsequently megalin-bound Aβ levels in CSF are reduced in AD patients, which may contribute to elevated brain Aβ. Finally, we also suggest that this effect might be specific to AD and not to other neurodegenerative disorders, such as PD. However, further studies using large cohorts are needed to confirm this last hypothesis.

## Conflict of Interest Statement

The authors declare that the research was conducted in the absence of any commercial or financial relationships that could be construed as a potential conflict of interest.
